# Promoting reusable and open methods and protocols (PRO-MaP) can improve methodological reporting in the life sciences

**DOI:** 10.1371/journal.pbio.3002835

**Published:** 2024-09-19

**Authors:** Sofia Batista Leite, Matthew A. Brooke, Annamaria Carusi, Andy Collings, Pierre Deceuninck, Jean-François Dechamp, Bronwen Dekker, Elisa De Ranieri, Emma Ganley, Annalisa Gastaldello, Fanglian He, Marcel LaFlamme, Ingrid Langezaal, James Morris, David Pamies, Monica Piergiovanni, Bernd Pulverer, David Sadler, Caroline Shamu, Vivian Siegel, Marco Straccia, Tracey L. Weissgerber

**Affiliations:** 1 European Commission, Joint Research Centre (JRC), Ispra, Italy; 2 NC3Rs, London, United Kingdom; 3 InterChange Research, Bloomsbury, London, United Kingdom; 4 eLife, Cambridge, United Kingdom; 5 European Commission, DG Research and Innovation (DG-RTD), Brussels, Belgium; 6 Nature Protocols, Springer Nature, Berlin, Germany; 7 Cell Press, Milan, Italy; 8 protocols.io, Springer Nature, Perth, United Kingdom; 9 Bio-protocol, Sunnyvale, California, United States of America; 10 PLOS, San Francisco, California, United States of America; 11 Science Europe, Brussels, Belgium; 12 Department of Biomedical Science, University Lausanne, Lausanne, Switzerland; 13 EMBO Press, Heidelberg, Germany; 14 F1000 Research, London, United Kingdom; 15 Harvard Medical School, Boston, Massachusetts, United States of America; 16 Department of Biology, Massachusetts Institute of Technology, Cambridge, Massachusetts, United States of America; 17 FRESCI by Science&Strategy SL, Barcelona, Spain; 18 Berlin Institute of Health at Charité – Universitätsmedizin Berlin, QUEST Center for Responsible Research, Berlin, Germany

## Abstract

Essential details about study methods are often missing from academic research papers in the life sciences, which can adversely affect reproducibility, undermine trust and impair researchers’ ability to reuse methods and data. This Perspective article describes PRO-MaP (Promoting Reusable and Open Methods And Protocols), which aims to increase and improve the reporting of detailed, structured and open methods and reusable step-by-step protocols in the life sciences.

## Teaser

Detailed method descriptions are essential for reproducibility, research evaluation, and effective data reuse. We summarize the key recommendations for life sciences researchers and research institutions described in the European Commission PRO-MaP report.

The open science movement has heavily focused on Open Access publications, and open and FAIR (Findable, Accessible, Interoperable, Reusable) data and code; however, the development of open methods has received comparatively little attention. An active community is needed to advance open, reusable methods and step-by-step protocols, as these research outputs are crucial for several reasons. First, reproducibility starts with methods; researchers cannot reproduce research findings or determine whether they are trustworthy without knowing how the data were generated [1]. Second, detailed methods are also crucial for responsible and effective data reuse, as they allow prospective data users to determine whether existing data sets are appropriate to answer new research questions, and whether the data were collected using a rigorous design that is likely to yield trustworthy and reproducible results. Third, in many fields, methods may be one of the most useful and reusable outputs that researchers create. Sharing methods, and giving credit to method developers, may accelerate scientific advancement. Unfortunately, the methods section of many research articles remains insufficient to reproduce results or reuse methods [2,3].

The Promoting Reusable and Open Methods and Protocols (PRO-MaP) recommendations [4] seek to address this problem by improving the reporting of detailed, reusable, and open methods and step-by-step protocols in the life sciences. PRO-MaP outlines actions that 4 stakeholder groups—researchers, research institutions and departments, publishers and editors, and funders—should take to achieve these goals. The recommendations focus on reusable step-by-step protocols, which describe how a specific procedure is performed, rather than study design protocols, which describe the research plan for a single study. PRO-MaP was conceptualized during a workshop convened by the EU Reference Laboratory for Alternatives to Animal Testing (EURL ECVAM, https://joint-research-centre.ec.europa.eu/eu-reference-laboratory-alternatives-animal-testing-eurl-ecvam_en) to bring together members of all stakeholder groups in June 2022. Recommendations were revised after receiving feedback from many members of each stakeholder group.

Readers can find complete, detailed recommendations for each stakeholder group in the full European Commission “Science for Policy” report [4], along with actions for implementing each recommendation. Here, we highlight important principles, and corresponding recommendations, for researchers and research institutions and departments (Table 1).

**Table 1:** PRO-MaP recommendations for researchers and research institutions and departments.

Stakeholder group	Key recommendations*
Researchers	Document, share and follow detailed protocols within your research group Follow study design and reporting guidelines (e.g., [5–7]) when designing and conducting your studies and reporting results Describe methods in enough detail to allow others to reproduce the experiments Ensure availability of methods and materials reported in papers and publications Support a research culture that rewards and incentivizes methods development and protocol sharing
Research institutions and departments	Create an environment that recognizes the value of sharing open and reproducible methods Reward and incentivize sharing of detailed methods and step-by-step protocols Require and offer training on writing and openly sharing detailed methods and reusable step-by-step protocols Integrate sharing of detailed methods and reusable step-by-step protocols into thesis requirements Monitor practices and obtain feedback on activities to encourage sharing of methods and reusable step-by-step protocols

* The full report [4] outlines actions that stakeholders can take to implement each recommendation.

**Share reusable step-by-step protocols and cite them in publications.** Formulating methods as step-by-step procedures makes it easier to identify and supply information that is essential to implement and reproduce the work. This is of greater value than the free text descriptions used in many research papers, which only provide a general overview of the methods used. As described above, institutions and departments should incentivize and reward researchers for sharing detailed methods and reusable step-by-step protocols by including these outputs in research(er) evaluations. Research group leaders, institutions, and departments should also offer training, including in the use of study design and reporting guidelines (e.g., [5–7], https://www.equator-network.org), using research resource identifiers (RRIDs) to unambiguously specify what reagents and organisms were used [8] (https://scicrunch.org/resources), techniques for writing reusable step-by-step protocols and depositing them in online repositories, and techniques for citing protocols in research papers.

**Protocols should be citable and shared on dynamic platforms.** While static method and protocol descriptions are essential for understanding (published) results, they reflect one researcher’s approach at a single time point and may quickly become outdated. Dynamic protocol sharing platforms, such as protocols.io [9], better capture the ever-changing reality of methods by allowing researchers to share updated versions of their protocol, or forks containing their adaptation of protocols from others. Each protocol object (with a DOI) represents the static protocol version used for a specific study; versioning and forking allows researchers to create new citable objects that more accurately reflect the methods used in their current experiments, while citing the static version of the protocol. Best practice is for researchers to both capture reusable step-by-step protocols detailing their research procedures and to use and maintain up-to-date versions of them. Research institutions and departments should provide training and reward researchers for publicly sharing and citing reusable protocols.

**Use methodological shortcut citations responsibly.** Researchers use a methodological shortcut citation when they cite another resource that used the method, instead of fully describing the method [10]. Shortcut citations are used to explain how something was done and may be accompanied by phrases like “briefly” or “as previously described.” However, shortcut citations can seriously impair understanding of the method if the resource cited is missing details needed to implement the method, also uses a shortcut citation, or is not accessible for everyone to read. When determining whether to cite a resource as a shortcut, researchers should confirm that the cited resource: (1) provides a detailed, reusable description; (2) describes the method used in the citing study; and (3) is Open Access [10]. Resources that don’t meet these criteria can be cited to give credit to the methods’ creators; however, should not be cited as shortcuts. Authors should either fully describe the method or find or create another resource that meets the criteria.

**Facilitate cultural change.** We need a cultural shift to reward and incentivize methods development and sharing of reusable, open methods and protocols. Rewarding open protocols, data and code alongside traditional publications is especially important; until this is achieved, researchers who share these valuable outputs will be doing more work without recognition. Research institutions and departments must incentivize cultural change by creating an environment that recognizes the value of sharing open and reproducible methods, and rewarding and incentivizing sharing of detailed methods and step-by-step protocols. Institutions, departments, and research group leaders can all disseminate the recommendations within their network. Institutions and departments may add a methods and protocols section to CV templates, include methods and protocol sharing in hiring, promotion and tenure evaluations, and make sharing and reporting of step-by-step protocols a thesis requirement.

While this article focuses on researchers and research institutions and departments, other stakeholders are also critical to incentivizing high-quality methods reporting. PRO-MaP highlights several actions that funders can take to reward and incentivize methods sharing, including providing resources to ensure that researchers have the capacity to do this additional work, recognizing methods and protocols as valued research outputs, and requiring sharing of methods and reusable step-by-step protocols from funded work. Publishers and editors can also facilitate, reward, and incentivize methods and protocol sharing when assessing and disseminating research papers. Ensuring that readers can find detailed methods that were used to generate data in published work is crucial to assess the quality of the work and to meet the research community’s needs.

The PRO-MaP authors welcome contributions and collaborations with stakeholders working to implement these recommendations. We seek to build a community where individuals and organizations can develop a shared multi-stakeholder action plan, learn from each other’s experiences, and work collaboratively to drive cultural change. We encourage researchers, as well as leadership and administrative staff in research institutions and departments, to work collaboratively to begin implementing the PRO-MaP recommendations. Individuals, research groups and organizations can start with 1 or 2 items that would be easy to implement, while exploring opportunities to implement more challenging items over time. Researchers and research institutions and departments can also encourage funders, publishers, and editors to implement the recommendations, as transformative change will require collaborative action across all stakeholder groups. We encourage everyone who is interested in working towards PRO-MaP implementation, exploring opportunities to adapt these recommendations to suit the needs of other research fields, and building a community to reward and incentivize sharing of open and reusable methods and protocols, to contact the report authors. The lack of detailed methods and protocols is a major impediment to reproducibility. We must work collaboratively to make research publications more reusable and reproducible.
